# Immunisation and preventive measures against mpox in pregnant women and children: A scoping review

**DOI:** 10.1371/journal.pgph.0007583

**Published:** 2026-11-06

**Authors:** Lamisa Rahman, Fahima Nasrin Eva, A. K. M. Ahsanuzzaman, Sadia Monami, Anindita Tabassum, Mohammad Hayatun Nabi, Mohammad Delwer Hossain Hawlader

**Affiliations:** 1 Public Health Promotion and Development Society, Dhaka, Bangladesh; 2 BRAC James P Grant School of Public Health, BRAC University, Dhaka, Bangladesh; 3 National Tuberculosis Control Programme, Directorate General of Health Services, Dhaka, Bangladesh; 4 Department of Laboratory Medicine, Continental Hospital PLC, Dhaka, Bangladesh; 5 Projahnmo Research Foundation, Dhaka, Bangladesh; 6 Department of Public Health, North South University, Dhaka, Bangladesh; 7 NSU Global Health Institute, North South University, Dhaka, Bangladesh; The University of Manchester, UNITED KINGDOM OF GREAT BRITAIN AND NORTHERN IRELAND

## Abstract

Mpox (monkeypox) poses disproportionate risks to pregnant women and children, yet evidence on immunisation and prevention in these groups remains limited. This scoping review mapped and synthesised existing evidence on vaccines and non-pharmaceutical preventive measures for mpox in pregnant women and children, to assess reported effectiveness, and to identify implementation barriers and facilitators. Peer-reviewed and Grey literature were searched in PubMed, Scopus, and Google for records published since 2008; databases were last searched on 19 September 2025. Screening and data extraction followed a pre-specified protocol and the Preferred Reporting Items for Systematic Reviews and Meta-Analyses extension for Scoping Reviews (PRISMA-ScR) framework. Key themes included immunisation strategies, preventive measures, effectiveness, barriers, and facilitators. Forty-eight peer-reviewed articles and 16 Grey literature sources were included. Third-generation non-replicating vaccines (MVA-BN/JYNNEOS/Imvanex/IMVAMUNE) were consistently recommended for pregnant women and children because of their favourable safety profiles. Observational and programmatic estimates of vaccine effectiveness (single dose 36–86%; two doses up to approximately 89%) came almost entirely from adults, and no effectiveness estimate specific to pregnant women or children was identified. No source reported vaccine use by trimester, and paediatric data were limited to small studies. Ring vaccination and post-exposure prophylaxis (PEP) were supported mainly by adult data. Major barriers included limited vaccine supply and cold-chain and logistical constraints in low- and middle-income countries (LMICs), vaccine hesitancy driven by safety concerns, particularly in pregnancy, and the absence of randomised trials in pregnant women and children. Few sources were centred on Africa or other LMICs. Facilitators included international coordination, targeted surveillance, flexible delivery strategies such as intradermal dosing, and integration with maternal and child health services. Priorities include prospective safety and immunogenicity studies in pregnancy and childhood, equitable vaccine access in LMICs, and tailored risk communication to address hesitancy.

## Introduction

Monkeypox (mpox), a zoonotic disease caused by the monkeypox virus (MPXV) of the Orthopoxvirus genus, has emerged as a global public health concern following notable outbreaks in both endemic and non-endemic regions. Initially identified in humans in the Democratic Republic of Congo (DRC) in 1970, mpox was historically confined to Central and West Africa. The 2022 multi-country outbreak, however, involved widespread transmission across more than 100 countries, prompting the World Health Organisation (WHO) to declare a Public Health Emergency of International Concern (PHEIC) [1,2].

Transmission of mpox occurs through close contact with infected individuals, animals, or contaminated materials. The disease presents with fever, lymphadenopathy, and a characteristic rash. Although typically self-limiting, severe cases can lead to secondary infections, pneumonia, encephalitis, and sepsis [3,4]. Vulnerable groups, including pregnant women and children, face heightened risks of adverse outcomes because of altered and immature immune function, respectively [4,5]. In pregnancy, mpox has been associated with miscarriage, preterm birth, stillbirth, and congenital infection [6,7]. In children, the disease may be severe, and deaths have been reported, particularly in endemic settings [8,9].

Immunisation remains a cornerstone of mpox prevention. The Modified Vaccinia Ankara–Bavarian Nordic (MVA-BN) vaccine, marketed as JYNNEOS (USA), Imvanex (EU), and IMVAMUNE (Canada), and ACAM2000, originally developed for smallpox, offer cross-protection against mpox. JYNNEOS is preferred because of its superior safety profile, particularly in pregnant women and children, whereas ACAM2000 carries risks including foetal vaccinia and myocarditis [4,10]. Vaccination strategies include pre-exposure prophylaxis (PrEP) for high-risk groups and post-exposure prophylaxis (PEP) to mitigate disease severity. Global vaccine accessibility remains limited, however, especially in LMICs, thereby exacerbating existing health inequities [11].

Despite the availability of vaccines, evidence on their safety, immunogenicity and effectiveness in pregnant women and children remains limited. Most data are extrapolated from smallpox studies or observational findings from recent mpox outbreaks. For pregnant women, MVA-BN is recommended, as its non-replicating nature minimises foetal risk [4,12]. Its use in children, though generally considered safe, requires further research to validate effectiveness and address concerns regarding immune response variability [4,9]. The absence of randomised clinical trials in these populations represents a critical evidence gap [12].

Preventive measures beyond vaccination, including public health education, early diagnosis, and infection control practices, are also important for protecting these vulnerable groups. For pregnant women, strategies to reduce the risk of vertical transmission, such as heightened vigilance during delivery and the potential use of caesarean section, have been proposed [4,5]. For children, community-level interventions including hygiene practices and isolation protocols are emphasised [13].

This scoping review examines existing evidence on immunisation and preventive measures against mpox in pregnant women and children. By identifying knowledge gaps and synthesising current data, the review seeks to inform public health strategies and guide future research priorities to protect these at-risk populations.

## Methods

### Ethics statement

This scoping review was based entirely on published and publicly available sources and did not involve primary data collection from human participants; formal ethics committee approval and participant consent were therefore not required.

### Review protocol

A protocol specifying the study objectives, research questions, inclusion and exclusion criteria, and data sources was developed prior to conducting the review (Table 1). The review was conducted in accordance with the PRISMA extension for Scoping Reviews (PRISMA-ScR) checklist [14]. A completed PRISMA-ScR checklist is provided as a supplementary file (S1 Checklist).

**Table 1:** Scoping review protocol summary.

Component	Details
Objectives	1. To identify and map existing immunisation strategies and preventive measures for mpox in pregnant women and children. 2. To assess the effectiveness of these strategies in preventing mpox and related complications, and to identify barriers and facilitators affecting their implementation. 3. To provide recommendations for improving mpox prevention strategies based on the findings of the effectiveness assessment and barrier analysis.
Research questions	**Using the PCC (Population, Concept, Context) framework:** 1. What immunisation strategies and preventive measures have been implemented for mpox in pregnant women and children? 2. How effective are existing immunisation strategies and preventive measures in preventing mpox and related complications? 3. What barriers and facilitators affect the implementation of mpox prevention strategies? 4. What recommendations can be made to improve mpox prevention strategies based on the effectiveness and barrier analysis?
Inclusion criteria	Peer-reviewed literature: observational studies, systematic reviews, and narrative reviews on immunisation and preventive strategies for mpox in pregnant women and children. Grey literature: government policy documents, institutional reports, and guidelines from the WHO, CDC, UNICEF, and national health departments. Language: English. Geographic scope: global (no restriction).
Exclusion criteria	Commentaries, editorials, short communications, qualitative studies, and sources without available full text.
Timeframe	2008 to present (from first documented congenital mpox syndrome case) [6]. Databases were last searched on 19 September 2025.
Data sources	Peer-reviewed: PubMed, Scopus. Grey literature: Google (institutional websites of health ministries, WHO, and relevant programme-implementing organisations).

Abbreviations: CDC = Centers for Disease Control and Prevention; PCC = Population, Concept, Context; UNICEF = United Nations Children’s Fund; WHO = World Health Organisation.

### Eligibility criteria

Peer-reviewed studies were eligible for inclusion if they were observational studies, systematic reviews, narrative reviews, or other relevant reviews addressing immunisation and preventive strategies for mpox in pregnant women and children. Grey literature, including government policy documents, institutional reports, and guidelines from the WHO, CDC, UNICEF, and national health departments, was also included. All sources were required to be in English and were included regardless of geographical origin. The review timeframe began in 2008, coinciding with the first documented case of mpox in a pregnant person involving a stillborn foetus with congenital mpox syndrome [6]. Commentaries, editorials, short communications, qualitative studies, and sources without available full text were excluded. Qualitative studies were excluded because the review question addressed the uptake, safety and effectiveness of immunisation and preventive measures rather than experiences of them.

### Search strategy

Peer-reviewed literature was identified through systematic searches of PubMed and Scopus, last searched on 19 September 2025, using a structured Boolean strategy combining terms for the exposure (mpox/monkeypox/MPXV/Orthopoxvirus), population (pregnant women, children, paediatrics, neonates, infants), concept (immunisation, vaccination, vaccine coverage, preventive measures, infection control, isolation, quarantine, contact tracing, education campaigns), and outcomes (effectiveness, efficacy, risk reduction, barriers, facilitators). Grey literature was retrieved by hand-searching institutional and organisational websites including those of the WHO, CDC, UNICEF, FDA, and national health departments, last accessed on 19 September 2025. A PRISMA-ScR checklist was completed to ensure reporting transparency [14].

### Screening and data extraction

Duplicate removal was performed using Rayyan software [15]. Two researchers independently screened titles and abstracts, followed by full-text screening to determine final inclusion. Agreement between the two screeners was assessed on the 165 records screened during the screening phase for which both screeners’ independent decisions were documented, using the binary include or exclude decision. Observed agreement was 78.8% and Cohen’s kappa [16] was 0.48 (95% confidence interval 0.37 to 0.60), indicating moderate agreement according to the benchmarks of Landis and Koch [17]. Kappa was calculated on the independent decisions recorded before any discussion. Where the two screeners disagreed, they met to compare the reasons for including or excluding the record and reached a consensus decision. Where consensus could not be reached, the senior author reviewed the record and made the final decision. The same procedure was applied at full-text screening. Data extraction was performed using a unified template developed by the research team, with four researchers independently extracting data from each included study. The extraction template captured author(s), publication year, study design, geographic setting, and key findings related to the following themes: immunisation strategies; preventive measures; effectiveness of immunisation and preventive measures; barriers to implementation; and facilitators of implementation. Discrepancies were resolved through team discussion under the supervision of the senior investigator. The analytical framework comprising themes and sub-themes is presented in Table 2. Study-level characteristics for peer-reviewed studies and Grey literature are presented in S1 and S2 Tables respectively.

**Table 2:** Analytical framework: themes and sub-themes used for data extraction and synthesis.

Theme	Sub-theme
Mpox vaccination strategies for pregnant women and children	Vaccine types and safety profiles; immunogenicity, including comparative immune responses; efficacy, effectiveness and cross-protection against mpox; administration strategies (PrEP and PEP)
Preventive measures for mpox in pregnant women and children	Pre-exposure and post-exposure prophylaxis; infection control and surveillance; maternal and child health monitoring
Facilitators for effective mpox prevention	International collaboration and vaccine accessibility; healthcare infrastructure and public health support; monitoring systems and resource distribution
Barriers to mpox vaccine implementation	Vaccine hesitancy and safety concerns; logistical and infrastructure challenges; cultural and social barriers
Recommendations for improving mpox vaccination uptake	Education and counselling to reduce vaccine hesitancy; ensuring equitable vaccine distribution, especially in LMICs; advancing vaccine development and monitoring systems

Abbreviations: LMICs = low- and middle-income countries; PEP = post-exposure prophylaxis; PrEP = pre-exposure prophylaxis.

In this review, efficacy refers to protection measured in randomised trials, effectiveness to protection measured in observational or programme settings, immunogenicity to immune responses such as antibody titres, and safety to adverse events. Where a source used these terms differently, its findings are reported under the category that matches the outcome measured.

## Results

### Study selection and characteristics

Forty-eight peer-reviewed articles [18–65] and 16 Grey literature sources [66–81] on vaccination and mpox prevention for children and pregnant women were included in this scoping review (Fig 1). Included studies covered a wide geographic range. Classified by the country of the lead institution recorded in S1 Table, the largest groups were the United States (n  =  11), China (n  =  6), the United Kingdom (n  =  4), Spain (n  =  3) and Nigeria (n  =  3), with 2 each from Egypt, Germany, India, Iran and Pakistan and 1 each from Australia, Brazil, Canada, the Democratic Republic of the Congo, France, Italy, Japan, Jordan, Malaysia, Mexico, Saudi Arabia and Singapore. Many of the included sources were reviews drawing on data from several regions, so this classification reflects where the authors were based rather than where data were collected. By study design, the peer-reviewed literature comprised 29 reviews (including 6 systematic reviews, 4 of which included a meta-analysis), 10 cross-sectional studies, 6 cohort studies, 1 observational clinical study, 1 modelling study and 1 case report. Grey literature sources included WHO guidance documents, government guidelines, public health information documents, and surveillance reports. The characteristics of the peer-reviewed studies are given in S1 Table and those of the Grey literature sources in S2 Table.

**Fig 1:**
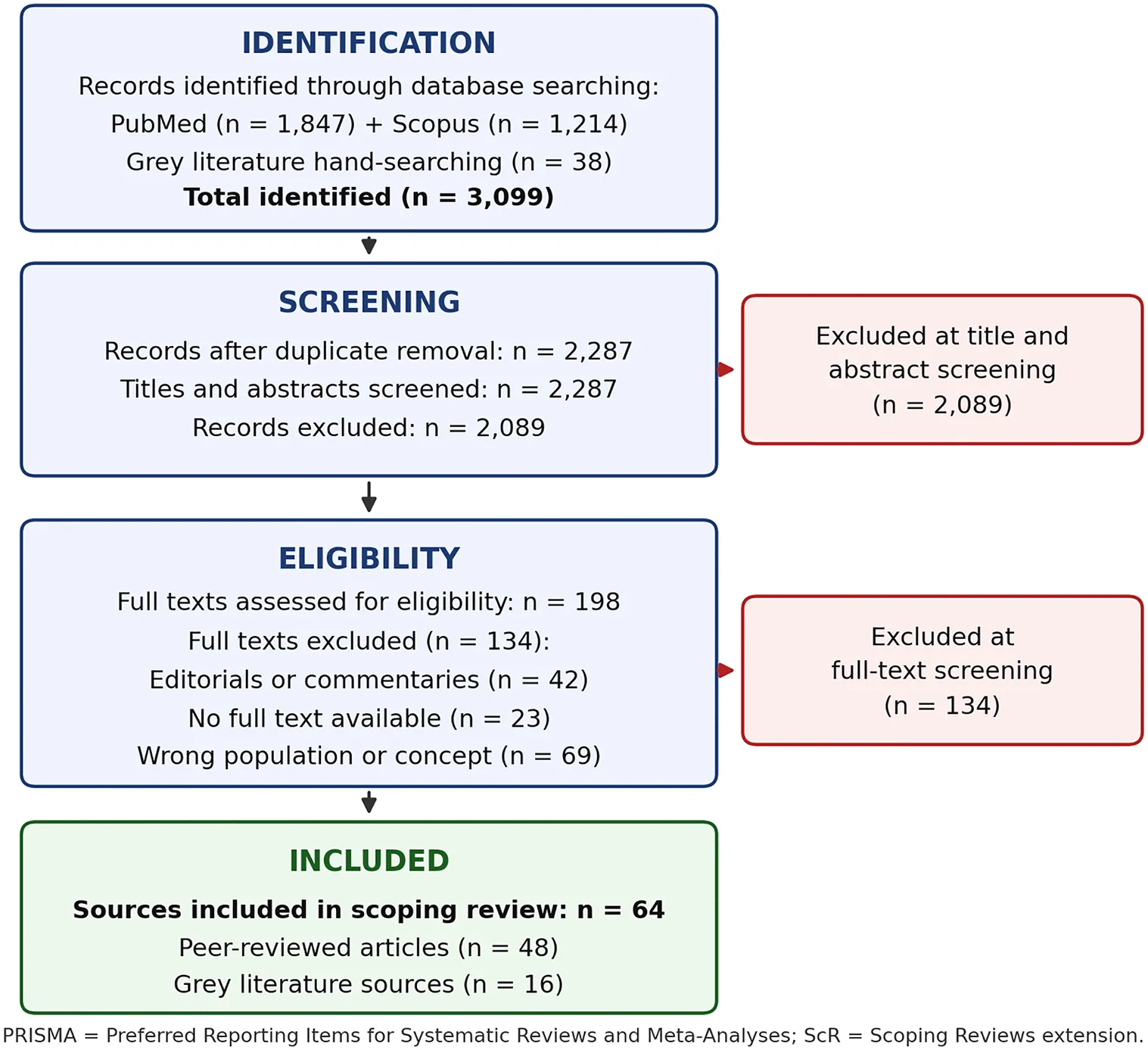
PRISMA-ScR flow diagram for study selection. PRISMA  =  Preferred Reporting Items for Systematic Reviews and Meta-Analyses; ScR  =  Scoping Reviews extension.

Most primary studies were conducted in the United States and Europe, and few sources were centred on Africa or other LMICs, although mpox was first described in humans in the Democratic Republic of the Congo and has been documented in central and west Africa for decades [18,19,21]. Five sources focused explicitly on these settings: an enhanced surveillance programme in the Democratic Republic of the Congo that addressed vaccination of health workers in endemic areas [27], a review of mpox in Nigeria [19], a survey of factors influencing vaccination in India [55], a review of vaccine access in the global south [63], and the WHO regional roadmap for mpox vaccination in Africa [67]. None of these reported primary data on the safety, immunogenicity or effectiveness of mpox vaccines in pregnant women or children.

### Immunisation strategies and effectiveness of vaccination

Third-generation smallpox vaccines, particularly MVA-BN (JYNNEOS/Imvanex/IMVAMUNE), represent the mainstay of immunisation for preventing mpox in children and pregnant women, because of their non-replicating nature and favourable safety profiles [25,27–34,36,66,69,76,80]. In contrast to second-generation vaccines such as ACAM2000, which contain live virus and are not recommended during pregnancy, MVA-BN has demonstrated acceptable safety and immunogenicity. JYNNEOS has demonstrated a superior humoral immune response compared with ACAM2000, and ACAM2000 is contraindicated in pregnancy and in infants because of the risk of adverse events [22,30]. Views on the minimally replicating vaccine LC16m8 differed between included sources: one review considered it another safe option during pregnancy and breastfeeding [24], whereas another reported that it is not recommended in pregnancy [46].

The effectiveness estimates in the included sources came almost entirely from adults. Single-dose estimates ranged from 36% to 86%, including 78% in England and 86% in Israel, and two-dose estimates reached approximately 89% among people without immunocompromise [25,32,74]. Where the study population was described, it consisted of adults, mostly men who have sex with men, during the 2022 outbreak [40,47]. Compared with unvaccinated individuals, MVA-BN vaccination was associated with a seven- to ten-fold reduction in mpox incidence in a cohort from Catalonia [33]. Several included reviews repeated an estimate of approximately 85% cross-protection for JYNNEOS and other smallpox vaccines [30,64], but this figure comes from surveillance of first-generation smallpox vaccination in central Africa in the 1980s and from later outbreaks in the same area [38], and it has not been measured for MVA-BN. No included source reported an effectiveness estimate for pregnant women or for children. The only paediatric data on MVA-BN came from a study in England of 87 children who received one post-exposure dose, none of whom developed mpox or a serious adverse event; that study assessed safety and immune response and was not designed to estimate effectiveness [25]. A systematic review of paediatric, maternal and congenital mpox found a similarly small evidence base: two studies reported vaccine safety in 28 recipients, seven reported maternal or congenital outcomes, and no published study of third-generation vaccine use in pregnancy was identified. The authors called for funding and equitable research in endemic countries [21]. Estimates from adults may therefore not apply to pregnant women or to children of different ages, whose immune function, exposure patterns and dosing may differ. Table 3 summarises what the included sources reported by pregnancy stage and by age group of children.

**Table 3:** Mpox vaccine use reported in the included sources, by pregnancy stage and age group of children.

Population group	Vaccine use and recommendations reported	Type of evidence	Sources
Pregnancy, stage not specified	MVA-BN preferred for pre-exposure and post-exposure vaccination because it is non-replicating. ACAM2000 contraindicated because of the risk of foetal vaccinia and foetal death. LC16m8 described as an alternative option in one review and as not recommended in another. Post-exposure vaccination advised within four days of exposure. A pregnancy test before vaccination was advised, and vaccination was not considered a reason to end a pregnancy.	Narrative and systematic reviews; WHO, FDA and health department guidance. No primary study of an mpox vaccine in pregnancy.	[20–22,24,26,30,46,66,68,76,77,80]
First, second or third trimester	No included source reported vaccine use, safety or effectiveness by trimester.	None identified	Not applicable
Breastfeeding and postpartum	MVA-BN and LC16m8 were described as options during breastfeeding in one review. JYNNEOS was recommended for eligible people who are pregnant, recently pregnant or breastfeeding. Temporary suspension of breastfeeding was advised for people with active lesions, to protect the infant.	Narrative review; MMWR surveillance report; case report	[24,49,80]
Newborns and infants under 1 year	Newborns exposed to mpox should be vaccinated, according to one review. ACAM2000 is contraindicated in infants. No included source reported MVA-BN outcomes specifically for infants.	Narrative and comparative reviews	[22,26]
Children (median age of questionnaire respondents 5 years)	One dose of MVA-BN as post-exposure prophylaxis in 87 children exposed to a confirmed case in England (June to November 2022). No serious adverse events and no mpox after vaccination were reported. Immune responses were measured at one and three months. Some parents declined vaccination.	Observational study; not designed to estimate effectiveness	[25]
Children and adolescents, age band not specified	MVA-BN described as suitable for children in several reviews, and emergency use authorisation existed for children in some regions. LC16m8 is licensed for children in Japan and has more paediatric data. The FDA fact sheet for JYNNEOS described use in people aged 18 years or older. The WHO regional roadmap for Africa listed children among priority populations.	Reviews; guidance documents	[21,22,46,47,67,76]
Effectiveness by age group or pregnancy stage	No included source reported an effectiveness estimate for any group under 18 years or for pregnant women. Published estimates were from adults.	Not available	[32,40,47]

Dose-sparing strategies, including intradermal MVA-BN delivery, have been investigated without apparent compromise of immunogenicity or effectiveness in adults [47,68]. Ring vaccination targeting close contacts of confirmed cases has been described as an effective way to contain outbreaks by establishing localised immunity [31]. A single dose of MVA-BN administered within 14 days of exposure has been reported to substantially limit mpox development in adults, though breakthrough cases have been documented, particularly among individuals with repeated sexual exposure [40]. The recommended regimen for pregnant women and children is two doses of MVA-BN [66,68], and one included review described one-, two- and three-dose strategies depending on exposure risk and vaccine availability [34]. Case-control studies in the United States, summarised in a systematic review, reported MVA-BN effectiveness of 36% to 89% depending on dose and immune status, with higher effectiveness in people without immunocompromise [32].

### Preventive measures for mpox in pregnant women and children

Both pharmaceutical and non-pharmaceutical approaches are used to prevent mpox in children and pregnant women. The non-replicating MVA-BN (JYNNEOS) vaccine remains the primary protective intervention and is recommended over ACAM2000 because of its superior safety profile for immunocompromised individuals, including children and pregnant women [77]. Multiple studies emphasise the importance of PEP administered four to 14 days after exposure and pre-exposure vaccination (PrEP) for high-risk groups, including healthcare workers in endemic areas [27,28,34,40,43]. Ring vaccination targeting close contacts of confirmed cases has shown effectiveness in establishing localised immunity [23]. Emerging strategies including nanotechnology-based vaccine delivery systems have been investigated to enhance immunogenicity while reducing side effects [29,30].

Beyond vaccination, infection control measures including stringent isolation of ill patients, maintenance of high hygiene standards, and use of personal protective equipment (PPE) by healthcare professionals are considered important for reducing nosocomial and community transmission [80]. Enhanced surveillance, encompassing rapid diagnostic testing, wastewater monitoring, and systematic contact tracing, has been identified as important for early epidemic detection and containment [40].

Non-pharmaceutical measures complement vaccination, particularly for susceptible groups. Public awareness programmes have been reported to be effective in educating communities about transmission risks and preventive measures [51]. Routine skin and mucosal examinations during pregnancy, together with serial ultrasound monitoring for foetal health assessment, are recommended to identify early infection signs [80]. Comparative data from various countries indicate that a combination of rigorous isolation, prompt vaccination, and public health education is associated with reduced mpox transmission [63]. Conversely, outbreaks tend to be prolonged in regions with limited public health infrastructure and restricted vaccine access. International efforts to integrate mpox vaccination into maternal and child health programmes require sustained support for LMICs in vaccine procurement and healthcare capacity-building [46,55,59–61,72]. Emerging antivirals including brincidofovir and tecovirimat (TPOXX) represent a developing field of post-exposure care that may be of benefit to high-risk individuals including pregnant women [20,31,56].

### Facilitators for mpox preventive strategies

Several factors are considered important facilitators of effective mpox prevention for pregnant women and children. International cooperation and regulatory support are central to vaccine accessibility, particularly regarding strategic stockpiles of ACAM2000 and JYNNEOS [28–30,36,54,59,62,63,79,80]. Enhanced surveillance systems enabling early case detection and targeted vaccination campaigns, including ring vaccination, have been supported by coordinated action from the CDC and WHO [23,27,37,57,64]. The adaptability of vaccine administration routes, including intradermal, subcutaneous, and intramuscular delivery, allows for more flexible distribution even in resource-limited settings [33,34,44,47,60,70,78]. Government support and public health initiatives have contributed to increasing awareness and improving vaccination uptake among high-risk groups [22,41,48,51]. Accessible healthcare infrastructure, including programmes for testing, treatment, and immunisation, has been identified as a key enabler, as demonstrated in settings with universal healthcare systems [33,37,44,49,58]. International cooperation, continuous research, and ongoing vaccine development further contribute to outbreak preparedness [18,24,38,40,42].

### Barriers to vaccine implementation

Vaccine hesitancy remains a substantial barrier, particularly among pregnant women concerned about possible foetal risks and side effects associated with vaccines such as ACAM2000, which has been linked to myocarditis and pericarditis [25,28–30,35,36,41,48,54,58,59,62]. Logistical obstacles, including limited healthcare infrastructure, vaccine shortages, and cold-chain challenges, complicate distribution in LMICs, especially in remote and underserved locations [23,31,37,41,44,57,60,61,63,71,72]. Social and health inequalities place marginalised groups at elevated risk, with reduced access to healthcare facilities and vaccines further exacerbated by disease misdiagnosis [34,49,50,55,80].

Vaccine scarcity and high cost further restrict access, particularly in low- and middle-income countries [22,28,29,53]. High-income countries secured large vaccine supplies early, whereas LMICs could not independently obtain substantial quantities [63], and WHO situation reports documented persistent gaps in vaccination coverage in these settings [71,72]. Limited diagnostic access in endemic areas was also reported to delay immunisation and case tracking [21]. Behavioural and cultural barriers, including vaccine hesitancy and misinformation, are apparent in communities with limited trust in healthcare systems [22,39,51,65]. The limited availability of population-specific data for pregnant women complicates the development of tailored prevention strategies and necessitates reliance on evidence extrapolated from non-pregnant populations [18–20,24,26,38,42,61]. Concerns about the long-term safety and effectiveness of newer vaccines including MVA-BN, and the risk of breakthrough infections, present additional challenges [21,24,40,45]. Variations in immune responses, including a limited B-cell response reported with JYNNEOS, raise questions regarding the adequacy of protection [34].

### Recommendations for improving vaccination uptake

The evidence supports several recommendations for improving vaccine uptake. Effective communication strategies targeting pregnant women and caregivers should address concerns about foetal risks and potential side effects [19,20,26,35,38,39,44,76]. Healthcare providers should be equipped to deliver supportive counselling to reduce hesitancy among high-risk populations [20,28,46–49]. Non-replicating vaccines such as MVA-BN are recommended for pregnant women in preference to replicating vaccines such as ACAM2000 [20,23,24,32,46].

Equitable global vaccine distribution, particularly in LMICs, remains a priority for protecting vulnerable populations including children, pregnant women, and healthcare workers [20,31,40,44,50]. Increasing vaccine production, enhancing cold-chain infrastructure, and prioritising high-risk groups in endemic areas, including children, are considered important for broadening coverage [18,21,22,33,40–42,47,51,54,60,67,71].

Expanding surveillance systems to monitor vaccine effectiveness and track outbreaks is considered essential for improving immunisation strategies and enabling early intervention [18,19,21,23,25,34,41,59,67]. Prioritising the development of vaccines with broader-spectrum efficacy, using technologies such as mRNA platforms and nanotechnology, may improve future vaccine options [29,30,37]. Vaccine trials in diverse populations, including pregnant women, should be continued to validate safety and optimise dosing regimens [26,37,40,42,43,45]. Universal adoption of standardised vaccination protocols prioritising high-risk populations is considered important for improving outbreak management and reducing transmission [31,40,52,53,58].

## Discussion

This scoping review provides a synthesis of immunisation strategies and preventive measures for mpox in pregnant women and children, drawing on evidence from 48 peer-reviewed articles and 16 Grey literature sources. The findings support the value of vaccination but reveal important evidence gaps and implementation barriers that may limit worldwide prevention effectiveness. Effectiveness data came almost entirely from adults, and no included source provided trimester-specific information.

The available literature indicates that MVA-BN elicits measurable immune responses and has been deployed widely during recent outbreaks, but antibody kinetics and durability vary by dose and prior orthopoxvirus exposure, reflecting uncertainty about optimal schedules for vulnerable groups [82,83]. Clinical guidance and expert reviews recommend non-replicating vaccines for use during pregnancy because animal data and mechanistic considerations suggest lower foetal risk compared with replicating vaccines; human pregnancy safety data remain limited and largely observational, however [4,12,81]. Surveillance reports and case series documenting adverse pregnancy outcomes after mpox infection, including miscarriage and stillbirth, reinforce the public health imperative to protect pregnant individuals through both prevention and early clinical management [5,7].

Real-world effectiveness estimates from outbreak settings suggest substantial, though variable, protection after one or two doses of MVA-BN, with single-dose effectiveness reported in some analyses in the mid-70% range and higher protection observed following completion of the two-dose series; interpretation is complicated by differences in study design, population risk heterogeneity, and potential confounding [47,84,85]. Vaccination has also been associated with milder disease among breakthrough cases [52,86]. Comparative safety concerns for replicating vaccines such as ACAM2000, including documented myocarditis and pericarditis risk and contraindications in pregnancy, support current recommendations to avoid their use in pregnant and immunocompromised individuals [10]. Ring vaccination and timely PEP have shown effectiveness as outbreak containment tools in several settings, but their success is contingent on rapid case detection and contact tracing capacity [87].

These estimates should not be applied directly to pregnant women or children. They were generated in adults, mainly men who have sex with men, during the 2022 outbreak, and current United States recommendations for routine JYNNEOS use refer to people aged 18 years or older [47,88]. Pregnancy alters immune responses and the immune system of young children is still maturing, so antibody kinetics, reactogenicity and the number of doses needed may differ from those in adults. Exposure patterns may also differ between adults in the 2022 outbreak and children and pregnant women in endemic settings. The paediatric study identified reported safety and immune responses but no effectiveness [25], and no source reported vaccine outcomes by trimester. Until such data exist, recommendations for these groups largely rest on the non-replicating nature of MVA-BN, animal data and adult effectiveness, and are best read as precautionary rather than evidence-based.

Implementation challenges are central to translating vaccine effectiveness into population-level impact. Supply constraints, cold-chain and logistical barriers in LMICs, and vaccine hesitancy driven particularly by pregnancy safety concerns have limited uptake and contributed to inequitable protection [26,35,63,71,72]. Operational adaptations such as intradermal dose-sparing and integration of mpox vaccination into maternal and child health platforms show promise, but require further validation of immunogenicity and programmatic feasibility across diverse contexts [61,82]. Strengthening surveillance, standardising outcome definitions, and expanding prospective safety monitoring in pregnancy and paediatrics are considered critical priorities for reducing uncertainty and guiding policy [9].

Taken together, the evidence supports a layered prevention approach combining the preferential use of non-replicating MVA-BN vaccines with strong infection control, targeted PEP, ring vaccination where feasible, and culturally appropriate risk communication to address hesitancy and improve equity. Research priorities include prospective vaccine safety and immunogenicity studies in pregnant and paediatric cohorts, harmonised surveillance to enable pooled analyses, and implementation research to address logistical barriers in LMICs. Attention to these priorities would help narrow the evidence-to-policy gap and better protect vulnerable populations from mpox [4,7,12,81].

The evidence reviewed is weighted towards high-income settings, whereas mpox has been documented for longest in central and west Africa [18,19]. Most effectiveness and programme data came from the United States and Europe, and the estimates that do come from Africa concern smallpox vaccines used decades ago [38]. Sources centred on Africa and other LMICs described the problem mainly as one of access: high-income countries secured large supplies early [63], and supply shortfalls, cold-chain constraints and limited diagnostic capacity slowed immunisation in endemic areas [21,71,72]. This is consistent with earlier accounts of unequal access to new vaccines in LMICs [11], and with the emphasis in the WHO regional roadmap on cold-chain capacity and on prioritising high-risk groups, including children [67]. The paediatric and maternal evidence from endemic settings is largely descriptive, and a systematic review found no published data on third-generation vaccines in pregnancy [21]. A recent commentary set out public health priorities for these groups in the Democratic Republic of the Congo [5]. A clinical trial of MVA-BN in pregnant and breastfeeding women and in children aged two years or younger was announced for the Democratic Republic of the Congo in November 2024 [89], and a protocol for the maternal trial has since been published [90]. Results from these trials will be needed before recommendations can rest on direct evidence. These findings suggest that global recommendations for pregnant women and children will remain provisional until safety and effectiveness data are generated in the settings where the disease burden is highest.

### Strengths and limitations

This review followed PRISMA-ScR methods and a pre-specified protocol and combined peer-reviewed and Grey literature, giving a broad, policy-relevant picture across regions. Several limitations apply. As a scoping review, it did not appraise study quality, and the evidence base was dominated by reviews and observational designs, many drawing on the same primary studies. Effectiveness estimates came from adult populations, and no source reported trimester-specific data. Evidence from Africa and other LMICs was sparse, and the restriction to English may have excluded relevant local sources. Qualitative studies were excluded, so the perspectives of pregnant women, caregivers and health workers are captured only indirectly. Inter-reviewer agreement at screening was moderate (kappa 0.48) and was assessed on a subset of records; disagreements were resolved by discussion, but some subjectivity in source selection remains. Finally, the search ended on 19 September 2025. Later evidence, including the protocol for the MVA-BN trial in pregnant women in the Democratic Republic of the Congo, is mentioned in the Discussion for context only and was not charted.

## Conclusion

This scoping review synthesises current evidence on immunisation and preventive measures for mpox in pregnant women and children. The balance of evidence supports the preferential use of non-replicating MVA-BN vaccines in these populations and highlights the potential value of combined pharmaceutical and non-pharmaceutical interventions, including PEP, ring vaccination, isolation, surveillance, and education. Persistent evidence gaps, particularly regarding high-quality safety, immunogenicity and effectiveness and efficacy data in pregnant and paediatric cohorts, including by trimester and age group and systemic barriers to equitable vaccine access, require attention as a matter of priority. Coordinated international action, strengthened surveillance, targeted research, and context-sensitive communication strategies are important for protecting pregnant women and children from mpox and for improving global preparedness for future outbreaks.

## Supporting information

S1 Checklist PRISMA-ScR checklist for this scoping review. (DOCX)

S1 Table Summary of peer-reviewed studies included for exploring immunisation and preventive measures against mpox in pregnant women and children (n = 48). (DOCX)

S2 Table Summary of Grey literature sources included for exploring immunisation and preventive measures against mpox in pregnant women and children (n = 16). (DOCX)

## Abbreviations

CDC: centers for disease control and prevention
DRC: Democratic Republic of Congo
EU: European union
FDA: U.S. food and drug administration
LMICs: low- and middle-income countries
MPXV: monkeypox virus
MVA-BN: modified vaccinia Ankara–Bavarian nordic
PCC: population, concept, context
PEP: post-exposure prophylaxis
PHEIC: public health emergency of international concern
PPE: personal protective equipment
PrEP: pre-exposure prophylaxis
PRISMA-ScR: preferred reporting items for systematic reviews and meta-analyses extension for scoping reviews
TPOXX: tecovirimat
UNICEF: United Nations Children’s Fund
USA: United States of America
WHO: World Health Organisation
