# Supplementary material for: Immunisation and preventive measures against mpox in pregnant women and children: A scoping review
Source: PLOS Glob Public Health. 2026 Nov 6;6(11):e0007583. doi: 10.1371/journal.pgph.0007583 (PMC13742946; doi:10.1371/journal.pgph.0007583)
Supplement: Supplementary file 1 [file pgph.0007583.s001.docx]

PRISMA-ScR Checklist

Preferred Reporting Items for Systematic Reviews and Meta-Analyses extension for Scoping Reviews (PRISMA-ScR)

Manuscript title: Immunisation and preventive measures against mpox in pregnant women and children: a scoping review

Journal: PLOS Global Public Health

| Section | PRISMA-ScR Item | Reported on page(s) | Details/Location in manuscript |
| --- | --- | --- | --- |
| Title | 1. Identify report as scoping review | 1 | Title |
| Abstract | 2. Structured summary | 2 | Abstract |
| Introduction | 3. Rationale | 3–5 | Introduction |
| Introduction | 4. Objectives | 5–6 | Introduction; Table 1 |
| Methods | 5. Protocol and registration | 5 | Methods; protocol developed, no registration reported |
| Methods | 6. Eligibility criteria | 7–8 | Eligibility criteria |
| Methods | 7. Information sources | 8 | Data sources; Search strategy |
| Methods | 8. Search | 8 | Search strategy; PubMed and Scopus last searched on 19 September 2025; grey literature last accessed on 19 September 2025 |
| Methods | 9. Selection of sources | 8–9 | Screening and data extraction |
| Methods | 10. Data charting process | 9 | Screening and data extraction |
| Methods | 11. Data items | 9–10 | Table 2 |
| Methods | 12. Critical appraisal | 24 | Not conducted as scoping review; Strengths and limitations |
| Methods | 13. Synthesis of results | 9–10 | Themes/subthemes, narrative synthesis |
| Results | 14. Selection of sources | 11–12 | Results; Fig 1 |
| Results | 15. Characteristics of sources | 11; Supplementary | Tables S1 & S2 |
| Results | 16. Critical appraisal within sources | 24 | Not applicable/not performed |
| Results | 17. Results of individual sources | 11–20; Supplementary | Narrative synthesis + Tables S1–S2 |
| Results | 18. Synthesis of results | 11–20 | Results sections |
| Discussion | 19. Summary of evidence | 20–25 | Discussion; Conclusion |
| Discussion | 20. Limitations | 24 | Strengths and limitations |
| Discussion | 21. Conclusions | 25 | Conclusion |
| Funding | 22. Funding | 26 | Funding statement |
