# Supplementary material for: Immunisation and preventive measures against mpox in pregnant women and children: A scoping review
Source: PLOS Glob Public Health. 2026 Nov 6;6(11):e0007583. doi: 10.1371/journal.pgph.0007583 (PMC13742946; doi:10.1371/journal.pgph.0007583)
Supplement: Supplementary file 2 [file pgph.0007583.s002.docx]

**Supporting Information**

**S1 Table. Summary of peer-reviewed studies included for exploring immunisation and preventive measures against mpox in pregnant women and children (n = 48).**

| **Reference number** | **Author(s), year** | **Title** | **Study design** | **Country/region** | **Intervention/topic** | **Outcome(s) assessed** | **Key findings** |
| --- | --- | --- | --- | --- | --- | --- | --- |
| 18 | Soheili M et al., 2022 | Monkeypox: virology, pathophysiology, clinical characteristics, epidemiology | Narrative review | Iran/global | Comprehensive review of mpox disease and prevention | Clinical management; prevention | Vaccination and surveillance key prevention pillars |
| 19 | Alakunle E et al., 2020 | Monkeypox virus in Nigeria: infection biology, epidemiology, and evolution | Narrative review | Nigeria | Epidemiological review including risk groups and prevention considerations | Epidemiology; prevention | Children identified as high-risk; vaccination and surveillance recommended |
| 20 | Dashraath P et al., 2022 | Monkeypox in pregnancy: virology, clinical presentation, and obstetric management | Narrative review | Singapore/global | Obstetric outcomes and management recommendations in pregnancy | Maternal and foetal outcomes | Adverse pregnancy outcomes documented; MVA-BN recommended; ACAM2000 contraindicated |
| 21 | Sanchez Clemente N et al., 2024 | Paediatric, maternal, and congenital mpox: systematic review and meta-analysis | Systematic review & meta-analysis | UK/global | Pooled analysis of paediatric, maternal, and congenital outcomes | Outcomes; prevention | Children and pregnant women at elevated risk; prevention remains a priority |
| 22 | Meo SA et al., 2022 | Comparison of JYNNEOS and ACAM2000 monkeypox vaccines | Comparative review | Saudi Arabia/global | Comparison of vaccine characteristics and safety | Safety; efficacy | JYNNEOS superior safety; recommended for immunocompromised including pregnant women |
| 23 | Kandeel M et al., 2023 | Efficacy of MVA vaccine and ACAM2000 in monkeypox prevention | Comparative review | Egypt/global | Head-to-head comparison of MVA-BN vs ACAM2000 | Efficacy; safety | MVA-BN superior safety; ACAM2000 not recommended in pregnancy |
| 24 | Daneji SM et al., 2022 | Monkeypox in human pregnancy: an overview | Narrative review | Nigeria | Review of pregnancy-specific mpox risks and prevention | Maternal outcomes | Adverse foetal outcomes documented; MVA-BN recommended for prevention |
| 25 | Ladhani SN et al., 2023 | Safety, reactogenicity, and immune response after single-dose MVA-BN in children | Clinical study (observational) | UK | Single-dose MVA-BN as post-exposure prophylaxis in 87 children exposed to a confirmed case (June to November 2022) | Safety; immunogenicity | No serious adverse events and no mpox after vaccination; immune responses assessed against unvaccinated paediatric controls; not designed to estimate effectiveness |
| 26 | Yan K et al., 2023 | Monkeypox and the perinatal period | Narrative review | China | Review of perinatal mpox risks and management | Maternal; neonatal outcomes | Heightened vigilance during delivery recommended; MVA-BN safest option in pregnancy |
| 27 | Petersen BW et al., 2019 | Vaccinating against monkeypox in DRC | Cohort study | DRC | Vaccination of health workers in an endemic area (Tshuapa Province) with a third-generation vaccine (IMVAMUNE), alongside CDC-supported enhanced surveillance | Healthcare-associated transmission; vaccination coverage | Healthcare-associated transmission has occurred repeatedly in endemic areas; vaccination of health workers proposed, with surveillance to document exposures |
| 28 | Reina J & Iglesias C, 2023 | Vaccines against monkeypox | Narrative review | Spain | Comparative review of available vaccines including MVA-BN and ACAM2000 | Safety; immunogenicity | MVA-BN preferred due to superior safety; ACAM2000 contraindicated in immunocompromised individuals |
| 29 | Saadh MJ et al., 2023 | Progress and prospects on vaccine development against monkeypox | Narrative review | Jordan/global | Review of vaccine pipeline and development progress | Vaccine development status | MVA-BN advances continue; mRNA platforms under investigation |
| 30 | Velázquez-Cervantes MA et al., 2022 | Mpox and pregnancy | Narrative review | Mexico | Review of maternal mpox outcomes and vaccine considerations | Maternal outcomes; vaccine recommendations | MVA-BN recommended in pregnancy; ACAM2000 contraindicated |
| 31 | Roper RL et al., 2023 | Mpox requires continued surveillance, vaccines, therapeutics, and mitigating strategies | Review | USA | Multi-pronged prevention including ring vaccination, PEP, and antivirals | Effectiveness; barriers | Combined strategies needed; supply barriers prominent in LMICs |
| 32 | Nave L et al., 2023 | Immunogenicity and safety of MVA vaccine: systematic review and meta-analysis | Systematic review & meta-analysis | China/global | Meta-analysis of randomised trials of MVA vaccine (safety and immunogenicity) | Safety; immunogenicity | No randomised trial evaluated clinical effectiveness; case-control studies cited in the review reported 36–86% (one dose) and 66–89% (two doses), higher in people without immunocompromise |
| 33 | Martín-Iguacel R et al., 2023 | Mpox: clinical outcomes and impact of vaccination | Cohort study | Spain | Clinical outcomes comparing vaccinated and unvaccinated patients | Hospitalisation; severity | Seven- to ten-fold reduction in incidence; hospitalisation 2% vs 8% |
| 34 | Ghazy RM et al., 2023 | Efficacy, effectiveness, safety, and immunogenicity of monkeypox vaccine: systematic review | Systematic review | Egypt/global | Pooled analysis of vaccine studies | Effectiveness; safety; immunogenicity | MVA-BN effective and safe; restricted B-cell activation noted with ACAM2000 |
| 35 | Liu S & Chu H, 2023 | Parents’ vaccination intention: COVID-19, HPV, and monkeypox | Cross-sectional | China | Survey of parental vaccine acceptance including mpox | Vaccine hesitancy; intention | Safety concerns principal driver of hesitancy; counselling recommended |
| 36 | Marzuki CNIFC et al., 2023 | Vaccines for orthopoxviruses: a review | Narrative review | Malaysia | Review of orthopoxvirus vaccines and cross-protection | Safety; cross-protection | MVA-BN preferred; mRNA platforms show promise |
| 37 | Malik S et al., 2023 | Recent developments in mpox prevention and treatment | Narrative review | Pakistan/global | Overview of prevention strategies and therapeutics | Prevention; treatment | Vaccination combined with antivirals recommended for high-risk groups |
| 38 | Poland GA et al., 2022 | Prevention of monkeypox with vaccines: a rapid review | Rapid review | USA/global | Assessment of available vaccine evidence | Efficacy; cross-protection | Approximately 85% clinical effectiveness of first-generation smallpox vaccines against mpox, from surveillance in central Africa in the 1980s and later outbreaks; MVA-BN preferred |
| 39 | Zheng Q et al., 2022 | Projecting impact of testing and vaccination on 2022 mpox outbreak in USA | Modelling study | USA | Epidemic modelling of vaccination coverage scenarios | Incidence reduction | Increasing vaccine coverage substantially reduces outbreak size |
| 40 | Merad Y et al., 2022 | Outcomes of post-exposure MVA vaccination to prevent mpox, Lyon, 2022 | Cohort study | France | PEP outcomes following contact tracing | PEP effectiveness | Single-dose MVA-BN within 14 days of exposure substantially limits disease development |
| 41 | Winters M et al., 2022 | Attitudes towards monkeypox vaccination among the US general public | Cross-sectional | USA | Survey of vaccination attitudes in US public | Vaccine hesitancy; intent | Safety concerns and lack of information principal barriers to acceptance |
| 42 | Lu J et al., 2023 | Mpox: pathogenesis, prevention, and treatment | Narrative review | China/global | Review of pathogenesis and prevention strategies | Prevention; treatment | MVA-BN recommended; antivirals for severe cases |
| 43 | Tomita N et al., 2023 | Safety and efficacy of smallpox vaccine LC16 as PEP for mpox | Cohort study | Japan | LC16m8 as PEP in exposed individuals | Safety; PEP effectiveness | LC16m8 safe and effective as PEP; may be considered during pregnancy |
| 44 | Zeien J et al., 2023 | Mpox in an urban homeless population and mobile vaccination clinic | Case series/cross-sectional | USA | Mobile vaccination in marginalised populations | Coverage; barriers | Mobile clinics improve equity; logistical and hesitancy barriers identified |
| 45 | Berry MT et al., 2024 | Predicting vaccine effectiveness for mpox | Cohort/modelling study | Australia/UK | Effectiveness modelling incorporating immune dynamics | Effectiveness prediction | Predicted effectiveness varies by prior exposure history; monitoring recommended |
| 46 | Grabenstein JD & Hacker A, 2024 | Vaccines against mpox: MVA-BN and LC16m8 | Review | USA/global | Comparative review of third-generation vaccines | Safety; efficacy | Both vaccines have solid safety records; MVA-BN non-replicating nature favours adoption, whereas LC16m8 has more paediatric data and is not recommended in pregnancy or immunocompromise |
| 47 | Pischel L et al., 2024 | Vaccine effectiveness of third-generation mpox vaccines: systematic review and meta-analysis | Systematic review & meta-analysis | Germany/global | Pooled effectiveness of third-generation vaccines | Effectiveness | Substantial effectiveness; intradermal dose-sparing non-inferior |
| 48 | Crosato V et al., 2024 | Perception and awareness about monkeypox and vaccine acceptance in Brescia, Italy | Cross-sectional | Italy | Survey of perceptions and acceptance among healthcare workers and public | Vaccine acceptance; hesitancy | Awareness positively associated with acceptance; education key facilitator |
| 49 | Lovett S et al., 2024 | Ocular mpox in a breastfeeding healthcare provider | Case report | USA | Clinical description of atypical mpox presentation | Clinical management | Highlights transmission risk in healthcare settings; vaccination of HCWs important |
| 50 | Andersen EW et al., 2024 | Mpox awareness, risk reduction, and vaccine acceptance among PLHIV in Washington DC | Cross-sectional | USA | Survey among people living with HIV | Vaccine acceptance; risk perception | Awareness high; structural barriers limit uptake despite intent |
| 51 | Oeser P et al., 2024 | The 5C model and mpox vaccination behaviour in Germany | Cross-sectional | Germany | Application of 5C psychological determinants model to mpox vaccination | Determinants of vaccine uptake | Confidence and complacency principal drivers; tailored communication recommended |
| 52 | Allard R et al., 2024 | Breakthrough mpox: one-dose vaccination associated with milder manifestations | Cohort study | Canada | Clinical severity in breakthrough cases following one-dose vaccination | Severity reduction | One-dose vaccination associated with milder disease even in breakthrough cases |
| 53 | Liu H et al., 2024 | Global perspectives on smallpox vaccine against monkeypox: comprehensive meta-analysis | Systematic review & meta-analysis | China/global | Pooled effectiveness across global vaccination programmes | Effectiveness | Cross-protection from smallpox vaccines substantial; two-dose MVA-BN superior |
| 54 | Wang X et al., 2024 | Current state and progress of mpox vaccine research | Narrative review | China | Review of vaccine landscape and development pipeline | Vaccine pipeline | MVA-BN current standard; LC16m8 and novel platforms under study |
| 55 | Rajkhowa P et al., 2023 | Factors influencing monkeypox vaccination: a cue to policy implementation | Cross-sectional | India/global | Survey-based assessment of vaccination determinants | Facilitators; barriers | Equity and access determinants vary by region; LMIC-specific strategies needed |
| 56 | Cabanillas B et al., 2023 | A compilation answering 50 questions on monkeypox | Review | Spain/global | Q&A synthesis covering epidemiology, prevention, and treatment | Prevention; management | Comprehensive synthesis supporting evidence-based prevention |
| 57 | Ghaseminia M, 2023 | Preventing monkeypox outbreaks: focus on diagnosis, care, treatment, and vaccination | Narrative review | Iran/global | Multi-component prevention review | Surveillance; prevention | Combined strategies including vaccination, surveillance, and treatment important |
| 58 | Christodoulidou MM & Mabbott NA, 2023 | Efficacy of smallpox vaccines against mpox in humans | Review | UK/global | Immunological basis of cross-protection | Immunogenicity; efficacy | Cross-protection demonstrated; mechanisms partially understood |
| 59 | Malone SM et al., 2023 | Safety and efficacy of post-eradication smallpox vaccine as mpox vaccine: systematic review | Systematic review | USA/global | Evidence on post-eradication vaccine use for mpox | Effectiveness; safety | Reasonable protection; adverse event profile acceptable; supply constraints noted |
| 60 | Owens LE et al., 2023 | JYNNEOS vaccination coverage among persons at risk for mpox: USA, May 2022–January 2023 | Cross-sectional/surveillance | USA | Coverage analysis of national JYNNEOS roll-out | Coverage; equity | Coverage improved over time; disparities by race and socioeconomic status noted |
| 61 | Piccolo AJL et al., 2023 | Critical elements of mpox vaccination model at the largest public health hospital in the USA | Cross-sectional/programme evaluation | USA | Evaluation of vaccination programme implementation | Programme elements; coverage | Integration with existing services and outreach facilitated uptake |
| 62 | Srivastava S et al., 2023 | The global mpox outbreak: a comprehensive review | Narrative review | India/global | Global outbreak review including prevention | Epidemiology; prevention | Vaccination central to control; global coordination required |
| 63 | Ogunkola IO et al., 2023 | Monkeypox vaccination in the global south: fighting a war without a weapon | Commentary/review | Nigeria/global | Equity analysis of vaccination access in LMICs | Equity; barriers | Severe supply shortfalls in LMICs; global solidarity mechanisms needed |
| 64 | Patel M et al., 2023 | Current insights into diagnosis, prevention, treatment, and challenges of mpox | Narrative review | Pakistan/global | Multi-component mpox review | Prevention; diagnosis | MVA-BN ~85% cross-protective; diagnostics and treatment improving |
| 65 | Torres TS et al., 2023 | Evaluation of mpox knowledge, stigma, and willingness to vaccinate among sexual and gender minorities | Cross-sectional | Brazil | Survey of knowledge and vaccine willingness in key populations | Willingness to vaccinate; stigma | Stigma barrier to care-seeking; knowledge associated with higher willingness |
| *Abbreviations: DRC = Democratic Republic of Congo; HCWs = healthcare workers; LC16m8 = LC16m8 smallpox vaccine; LMICs = low- and middle-income countries; mRNA = messenger ribonucleic acid; MVA-BN = Modified Vaccinia Ankara–Bavarian Nordic; PEP = post-exposure prophylaxis; PLHIV = people living with HIV.* | | | | | | | |
