# Supplementary material for: Immunisation and preventive measures against mpox in pregnant women and children: A scoping review
Source: PLOS Glob Public Health. 2026 Nov 6;6(11):e0007583. doi: 10.1371/journal.pgph.0007583 (PMC13742946; doi:10.1371/journal.pgph.0007583)
Supplement: Supplementary file 3 [file pgph.0007583.s003.docx]

**Supporting Information**

**S2 Table. Summary of Grey literature sources included for exploring immunisation and preventive measures against mpox in pregnant women and children (n = 16).**

| **Reference number** | **Source/author, year** | **Title** | **Document type** | **Geographic scope** | **Content relevant to review** | **Contribution to review themes** |
| --- | --- | --- | --- | --- | --- | --- |
| 66 | WHO, 2022 | Vaccines and immunization for monkeypox | Guidance document | Global | MVA-BN (JYNNEOS/Imvanex) recommended for high-risk groups including pregnant women; two-dose schedule recommended; ACAM2000 not recommended in pregnancy | Established MVA-BN as preferred vaccine; set international precedent for pregnancy guidance |
| 67 | WHO AFRO, 2024 | Mpox vaccination preparation roadmap | Roadmap/guidance | Africa region | Regional roll-out strategy for MVA-BN in Africa; prioritisation of high-risk populations including children | Highlighted LMIC equity gaps; outlined cold-chain requirements and priority populations |
| 68 | WHO, 2024 | MVA-BN (Modified Vaccinia Ankara–Bavarian Nordic) smallpox and mpox vaccine: interim guidance, 27 November 2024 | Guidance document | Global | Updated recommendations on MVA-BN administration routes, schedules, and populations including pregnant women and children | Confirmed safety in pregnancy; recommended two-dose schedule; endorsed intradermal dose-sparing approach |
| 69 | WHO, 2024 | Safety monitoring of mpox vaccines using cohort event monitoring: a WHO protocol | Protocol/guidance | Global | Protocol for active surveillance of adverse events following immunisation with mpox vaccines | Provided standardised methodology for safety signal detection across programmes |
| 70 | WHO, 2024 | WHO target product profiles for mpox vaccines, version 1 | Target product profile | Global | Defined preferred and minimally acceptable characteristics for mpox vaccines for both endemic and epidemic settings | Identified paediatric and maternal populations as key target groups for future vaccine development |
| 71 | WHO, 2024 | Mpox: multi-country external situation report no. 40 (October 2024) | Situation report | Global | Global situation update including case counts, outbreak spread, and vaccination coverage data | Documented ongoing gaps in LMIC vaccination coverage; reaffirmed vaccination as key containment measure |
| 72 | WHO, 2023 | Multi-country outbreak of mpox: external situation report 17 (February 2023) | Situation report | Global | Outbreak update including vaccination roll-out progress, coverage, and response challenges | Identified supply shortfalls and cold-chain barriers as principal LMIC challenges |
| 73 | NYC DOHMH, 2023 | JYNNEOS vaccine for mpox: frequently asked questions | FAQ/guidance | USA | JYNNEOS vaccine information including pregnancy and children guidance; ACAM2000 effectiveness estimate of 35.8% | Quantified lower effectiveness of ACAM2000; reinforced JYNNEOS preference in vulnerable groups |
| 74 | NYSDOH, 2022 | What to expect after your monkeypox vaccine | Public information | USA | Post-vaccination information including expected side effects and timeline; reported two-dose effectiveness approximately 89% | Quantified population-level effectiveness; communicated expectations to increase uptake |
| 75 | Vasan A / NYC DOHMH, 2022 | New York City Department of Health and Mental Hygiene mpox vaccination programme | Programme report | USA | Programme implementation report for mass mpox vaccination in New York City | Demonstrated operational model for urban mass vaccination; highlighted equity gaps by neighbourhood |
| 76 | FDA, 2024 | Fact sheet for recipients and caregivers about JYNNEOS (smallpox and mpox vaccine, live, non-replicating) | Regulatory document | USA | Official prescribing information; recommended for individuals ≥18 years at risk for smallpox or mpox; pregnancy category guidance included | Provided regulatory endorsement; maternal guidance based on animal safety data and non-replicating nature |
| 77 | LACDPH, 2023 | Mpox JYNNEOS vaccine: frequently asked questions | FAQ/guidance | USA | Community-facing vaccine information including eligibility and safety for pregnant and immunocompromised individuals | Strengthened public communication; reinforced JYNNEOS preference in vulnerable populations |
| 78 | NYSDOH, 2022 | Guidance for healthcare providers and vaccine administrators: monkeypox JYNNEOS vaccination information | Clinical guidance | USA | Provider-facing guidance on JYNNEOS administration routes including intradermal dose-sparing | Enabled dose-sparing approach at scale; provided clear clinical decision support for providers |
| 79 | NJDOH, 2022 | Frequently asked questions: monkeypox | FAQ/guidance | USA | State-level public information on mpox prevention, transmission, and vaccination | Supported community awareness; highlighted vaccination as primary prevention strategy |
| 80 | Oakley LP et al. / CDC, 2023 | Mpox cases among cisgender women and pregnant persons: United States, May–November 2022 | Surveillance report (MMWR) | USA | Epidemiological description of mpox cases in women and pregnant individuals; management and vaccination recommendations | Documented clinical presentations in pregnancy; reinforced MVA-BN recommendations; highlighted surveillance importance |
| 81 | WHO, 2025 | Clinical management and infection prevention and control for mpox | Clinical guidance | Global | Updated clinical management protocols including infection prevention and control recommendations for healthcare settings | Provided integrated guidance encompassing pharmaceutical and non-pharmaceutical prevention; referenced in Discussion for current best-practice context |
| *Abbreviations: CDC = Centers for Disease Control and Prevention; FDA = U.S. Food and Drug Administration; LACDPH = Los Angeles County Department of Public Health; LMICs = low- and middle-income countries; MMWR = Morbidity and Mortality Weekly Report; MVA-BN = Modified Vaccinia Ankara–Bavarian Nordic; NYC DOHMH = New York City Department of Health and Mental Hygiene; NJDOH = New Jersey Department of Health; NYSDOH = New York State Department of Health; WHO = World Health Organisation; WHO AFRO = WHO Regional Office for Africa.* | | | | | | |
