# Supplementary material for: Immunisation and preventive measures against mpox in pregnant women and children: A scoping review
Source: PLOS Glob Public Health. 2026 Nov 6;6(11):e0007583. doi: 10.1371/journal.pgph.0007583 (PMC13742946; doi:10.1371/journal.pgph.0007583)
Supplement: Supplementary file 4 [file pgph.0007583.s005.docx]

**Response to Reviewers**

Immunisation and preventive measures against mpox in pregnant women and children: a scoping review [Manuscript ID: PGPH-D-26-02250]

Dear Dr Janin and Dr Mutua,

Thank you for the helpful comments. We have addressed each one. Reviewer and editor comments are shown in italics, followed by our response and the page and line numbers in the revised manuscript. Page and line numbers refer to the clean version of the revised manuscript, which now carries continuous line numbering.

**Editorial requirements**

**E1.** *Data availability: include a minimal dataset and a statement that meets PLOS policy.*

**Response.** The Data Availability statement has been rewritten. For this scoping review the minimal data set is the data charted from every included source, which is presented in full in S1 Table (48 peer-reviewed studies) and S2 Table (16 Grey literature sources), together with Tables 1 to 3. All included sources are published and publicly available and are listed in the reference list, so no ethical or legal restrictions apply.

**Location.** p. 26, lines 482 to 487.

**E2.** *Place the ethics statement under a subheading at the start of the Methods.*

**Response.** Done. It now appears under the subheading Ethics Statement at the start of the Methods, and a corresponding statement is retained under Declarations.

**Location.** p. 5, lines 106 to 109.

**E3.** *Submit a response letter, a tracked-changes manuscript and a clean manuscript.*

**Response.** All three files are submitted, together with the revised figure and supporting files.

**E4.** *Figures must meet PLOS requirements and be processed with the NAAS tool.*

**Response.** Fig 1 was redrawn as a 300 dpi TIFF (1,650 × 1,500 pixels) and processed through NAAS. The content is unchanged except that the missing number for exclusions on grounds of wrong population or concept (n = 69) has been added, so the three reasons now sum to 134.

**Location.** Fig 1 file; p. 12, line 205.

**Reviewer 1**

**Comment 1.** *Reference 66 is missing from the reference list.*

**Response.** Numbering ran from 65 to 67 and no source or citation was attached to 66, so this was a numbering gap rather than a lost reference. We re-sequenced the whole list in order of first citation, so no numbers are now skipped. The included sources are numbered continuously, 18 to 65 for the peer-reviewed articles and 66 to 81 for the Grey literature, matching S1 and S2 Tables.

**Location.** References, p. 27 onwards.

**Comment 2.** *References 87 and 97 appear to be the same.*

**Response.** Correct, and they have been merged. Checking the whole list found eleven further duplicate pairs, which we also merged. The list now holds 90 unique references, numbered in order of first citation. A concordance of old and new numbers is available on request.

**Location.** References, p. 27 onwards.

**Comment 3.** *Capitalise Grey literature throughout and avoid "gery literature".*

**Response.** Done throughout the manuscript and supporting tables. We could not find the spelling "gery" and assume it was a slip in the comment.

**Location.** For example p. 2, line 37; p. 8, line 140.

**Comment 4.** *Global barriers and facilitators matter and are often reported in qualitative studies. A strong methodological justification is needed for excluding them.*

**Response.** We agree that qualitative evidence is important for understanding barriers and facilitators. We excluded qualitative studies for three methodological reasons. First, the review question was framed using the Population, Concept and Context structure recommended for scoping reviews, with the Concept defined as immunisation and preventive measures and their reported uptake, safety and effectiveness. This is an intervention and outcome question, and experiential accounts are not the evidence type that answers it. Second, qualitative evidence requires a different synthesis method, such as thematic or meta-aggregative synthesis with appraisal of methodological quality and of confidence in the synthesised findings. That approach differs from the descriptive charting used here, and combining the two in a single chart would risk presenting experiential themes and reported effectiveness as equivalent forms of evidence. Third, barriers and facilitators were still charted as reported in the eligible sources, which included surveys of vaccination intention and acceptability, programme evaluations, surveillance reports and national and international guidance, so these constructs are represented at population and programme level. We have added a one-sentence rationale to the Methods, and the limitations acknowledge that the accounts of pregnant women, caregivers and health workers are captured only indirectly. A dedicated qualitative evidence synthesis would be a valuable next step.

**Location.** p. 8, lines 130 to 132; p. 24, lines 444 to 445.

**Comment 5.** *Very little information is presented from Africa and LMICs.*

**Response.** We agree. Rather than adding a separate section, we have integrated this evidence into the relevant themes. Study selection and characteristics now identifies the five sources centred on Africa or LMICs and notes that none reported primary vaccine data for pregnant women or children. The effectiveness section reports the systematic review of paediatric, maternal and congenital mpox, and Barriers describes the inequity in vaccine access. The Discussion sets these findings against regional evidence, notes that the 85% figure often quoted comes from central African surveillance in the 1980s, and refers to the trial now under way in the Democratic Republic of the Congo. The scarcity reflects the evidence found, not a geographic restriction, as the review had none.

**Location.** p. 11 to 12, lines 193 to 201; p. 14, lines 234 to 238; p. 19, lines 321 to 324; p. 23 to 24, lines 419 to 435; p. 24, lines 443 to 444.

**Comment 6.** *Efficacy, effectiveness, immunogenicity and safety are used interchangeably in places.*

**Response.** We have added definitions of the four terms to the Methods and checked every use against them. Estimates are now reported as effectiveness with the population stated, and the meta-analysis of randomised trials is described as evidence on safety and immunogenicity only. We also corrected two statements that mixed categories: the 85% figure, which derives from older smallpox vaccines rather than JYNNEOS, and a single-dose figure attributed to ACAM2000, which we could not verify and have removed.

**Location.** p. 9, lines 167 to 170; p. 13, lines 221 to 230.

**Comment 7.** *Provide a table showing vaccine use across trimesters and across ages of children.*

**Response.** Table 3 has been added. It sets out vaccine use by pregnancy stage and by age group of children, with the type of evidence and the sources. It shows that no included source reported use, safety or effectiveness by trimester, and none reported effectiveness for any group under 18 years. We report these as evidence gaps.

**Location.** p. 14, lines 240 to 243 and the table following.

**Comment 8.** *General-population effectiveness of MVA-BN may not apply to pregnant women and children of different ages.*

**Response.** We agree and now say so directly. The Results state that the estimates came almost entirely from adults, mostly men who have sex with men during the 2022 outbreak, and that no source reported an effectiveness estimate for pregnant women or children. The only paediatric data came from one study of 87 children in England, which assessed safety and immune response only. The Discussion explains why adult estimates may not transfer.

**Location.** p. 13 to 14, lines 221 to 241; p. 24, line 442.

**Comment 9.** *Two researchers screened and four extracted data. State the statistical analysis used to measure inter-reviewer agreement.*

**Response.** We have calculated it. On the 165 records for which both screeners’ independent decisions were documented, observed agreement was 78.8% and Cohen’s kappa was 0.48 (95% confidence interval 0.37 to 0.60), which is moderate agreement on the Landis and Koch benchmarks. Cohen and Landis and Koch are now cited. We have also described the procedure in full: kappa was calculated on the independent decisions recorded before discussion; disagreeing screeners then met to compare their reasons and reach consensus; and where consensus could not be reached the senior author made the final decision.

**Location.** p. 8 to 9, lines 146 to 156; p. 24, lines 445 to 447.

**Other corrections made during revision**

While addressing the comments we checked the citations against our extraction sheet and corrected the following.

- The reference now numbered 32 (23 in the submitted version) was wrongly attributed. The systematic review of MVA safety and immunogenicity is by Nave et al. (Vaccines 2023), not Cheng et al. in Heliyon. The reference and the S1 Table entry have been corrected.
- Two reference details were corrected: the year for Velázquez-Cervantes et al. (2023) and the article number for Torres et al. (e46489).
- Citations throughout the manuscript were checked against the extraction sheet and reassigned where a cited source did not support the statement. This included the Facilitators, Barriers and Recommendations groups. Two figures that could not be traced to any included source were removed: a single-dose estimate attributed to ACAM2000, and a hospitalisation comparison (2% against 8%). All 90 references are cited in the text.
- The country and study-design counts were recounted from S1 Table and corrected (p. 11, lines 177 to 185).
- Continuous line numbering was added, and the PRISMA-ScR checklist page numbers were updated.
- The Introduction and Methods now cite only background literature, including two WHO sources added for the opening paragraph, and the Results cite only the included sources. A secondary news report cited for an effectiveness estimate was removed in favour of the primary sources.
- The Abstract was shortened to meet the 300-word limit.

We hope the revised manuscript is now suitable for publication and thank the editor and reviewer for their comments.

Sincerely,

Lamisa Rahman

Corresponding author, on behalf of all authors
